# European Society of Paediatric Radiology abdominal imaging task force recommendations in paediatric uroradiology, part IX: Imaging in anorectal and cloacal malformation, imaging in childhood ovarian torsion, and efforts in standardising paediatric uroradiology terminology

**DOI:** 10.1007/s00247-017-3837-6

**Published:** 2017-08-29

**Authors:** Michael Riccabona, Maria-Luisa Lobo, Lil-Sofie Ording-Muller, A. Thomas Augdal, E. Fred Avni, Johan Blickman, Constanza Bruno, Beatrice Damasio, Kassa Darge, Akaterina Ntoulia, Frederica Papadopoulou, Pierre-Hugues Vivier

**Affiliations:** 1 Department of Radiology, Division of Pediatric Radiology, University Hospital LKH Graz, Auenbruggerplatz 34, A-8036 Graz, Austria; 2 Department of Radiology, Hospital de Santa Maria-CHLN, University Hospital, Lisbon, Portugal; 3 Department of Radiology and Nuclear Medicine, Unit for Paediatric Radiology, Oslo University Hospital, Oslo, Norway; 4 Department of Radiology, University Hospital of North Norway, N-9038 Tromsø, Norway; 5 Department of Pediatric Radiology, Jeanne de Flandre Hospital, CHRU de Lille, Lille Cedex, France; 6 Department of Radiology, Golisano Children’s Hospital, Rochester, NY USA; 7 Radiology Institute, Department of Radiology, AOUI, Verona, Italy; 8 Department of Radiology, G. Gaslini Institute, Genoa, Italy; 9 Department of Radiology, The Children’s Hospital of Philadelphia, University of Pennsylvania, Philadelphia, PA USA; 10 Pediatric Ultrasound Center, Thessaloniki, Greece; 11 Radiologie, Hôpital Privé de l’ Estuaire, 505 rue Irène Joliot Curie, Le Havre, France

**Keywords:** Anorectal malformation, Child, Cloaca, Consensus, Diagnostic procedures, Fistolography, Fluoroscopy, Genitography, Guidelines, Imaging recommendations, Magnetic resonance imaging, Ovarian torsion, Pelvicalyceal dilatation, Terminology, Ultrasound

## Abstract

At the occasion of the European Society of Paediatric Radiology (ESPR) annual meeting 2015 in Graz, Austria, the newly termed ESPR abdominal (gastrointestinal and genitourinary) imaging task force set out to complete the suggestions for paediatric urogenital imaging and procedural recommendations. Some of the last missing topics were addressed and proposals on imaging of children with anorectal and cloacal malformations and suspected ovarian torsion were issued after intense discussions and a consensus finding process that considered all evidence. Additionally, the terminology was adapted to fit new developments introducing the term *pelvicalyceal dilatation/distension (PCD)* instead of the sometimes misunderstood *hydronephrosis.* The present state of paediatric urogenital radiology was discussed in a dedicated minisymposium, including an attempt to adapt terminology to create a standardised glossary.

## Introduction

The newly named European Society of Paediatric Radiology (ESPR) abdominal (gastrointestinal and genitourinary) imaging task force (formerly the Uroradiology task force) in cooperation with the European Society of Uroradiology paediatric working group set out to provide standards for imaging in paediatric urogenital radiology by issuing recommendations. These aim at harmonising imaging protocols, following the as low as reasonable achievable (ALARA) principle, and at reducing unnecessary invasiveness or burden.

The recommendations should help optimise imaging in childhood by providing procedural recommendations for the most relevant imaging methods, and imaging algorithms for all the typical queries in neonates, infants and children highlighting differences from the usual adult approach. The objective of diagnostic imaging should be to efficiently answer the clinically and therapeutically relevant questions at the lowest possible radiation burden. Finally, the task force wants to help create an environment where multi-institutional research and meta-analysis can be more easily performed to facilitate more evidence for the best imaging in neonates, infants and children.

These task force objectives were also outlined in a post-congress symposium, on the occasion of the 52nd ESPR annual meeting in Graz, Austria, on 6 June 2015, titled “Paediatric urogenital imaging today – the old and the new stuff for daily needs.” The purpose was not only to summarise, present and revisit the work of the task force over the past decade, but to offer a comprehensive update on paediatric urogenital radiology and present the context of the task force’s work.

- An initial clinical statement (“The Clinical Needs”) pointed out the importance of close cooperation with clinical colleagues and the need for a deep understanding of clinical needs when performing these studies – only then can the individually important queries be understood and answered in a way that reveals all necessary information for therapy and management decisions. As therapy options are changing, new strategies are being developed and new drugs are available, a constant refreshment and update of the (paediatric uro-)radiologists’ clinical knowledge is particularly important.
- Multiple procedures are addressed in the existing suggestions from the task force. These suggestions and their implications on practical daily needs were presented and discussed. The various imaging methods were revisited (“The Methods“), i.e. the procedural recommendations that shall help standardise imaging.
- The importance of clear documentation and proper structured reporting using a consistent terminology was highlighted (“The Report and the Terminology”); the respective proposal for a standardised glossary of paediatric uroradiological terms has been presented and is–after recent finalization of this multi-institutional and multidisciplinary consensus paper –under publication.
- The typical common diseases and scenarios in paediatric nephro-urology were listed and the potential of imaging in the respective conditions as well as their imaging appearances on the different modalities were presented (“The Diseases”).
- Imaging algorithms were addressed and the various recommendations of the task force were presented to try to address not only common conditions but also rarer, still important ones (“The Answers – Imaging Approaches”).
- Finally, potential future developments were discussed (“The Future – Necessary and Foreseeable Future Developments or Wishful Thinking?”) to try to imagine where the future will lead and how future developments will impact paediatric urogenital radiology. The risks and restrictions that may impair further development and improvement of paediatric urogenital imaging and its widespread use in children were also considered.

**Fig. 1:**
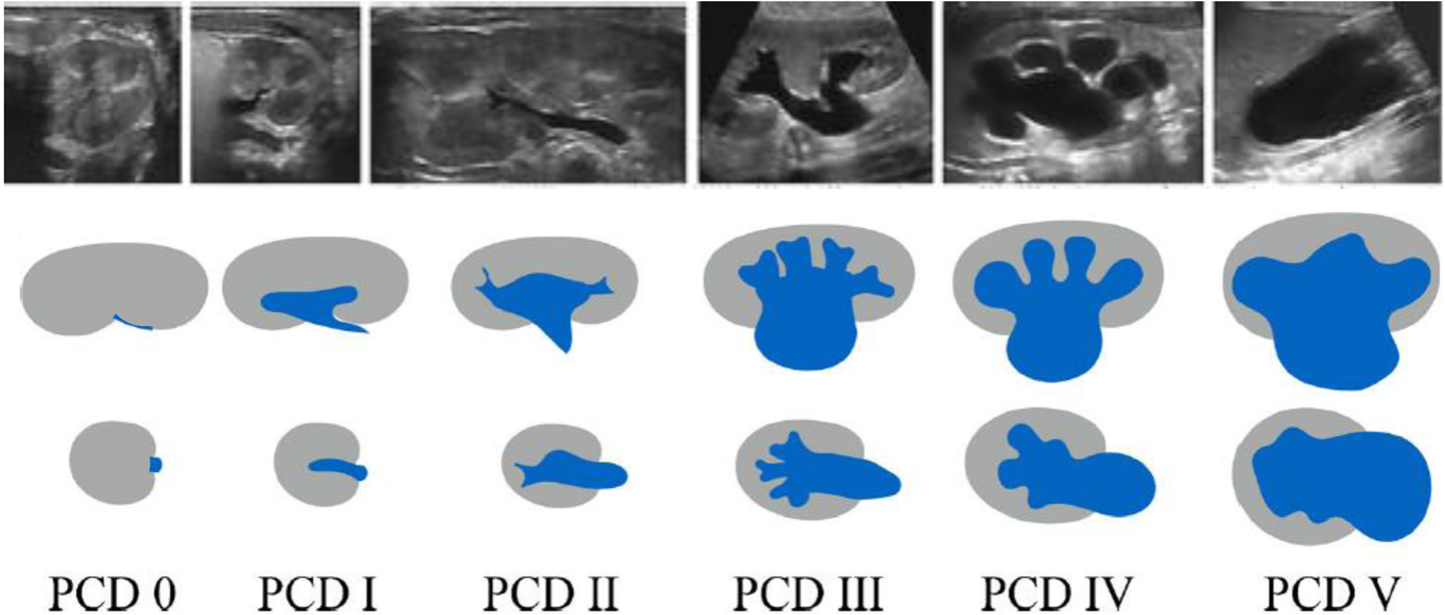
Sonographic grading of postnatal pelvicalycael distention/dilatation (PCD), formerly called the hydronephrosis grading system. *Top row*: longitudinal section. *Bottom row*: transverse section at the level of the renal pelvis. *PCR* 0 collecting system *PCD 0* collecting system not, or hardly, visible — normal; *PCD I* only renal pelvis clearly visible, calices not depictable, axial pelvic diameter<7 mm — considered normal; *PCD II* axial renal pelvis diameter<10 mm, (some) calices visible but with normal forniceal and papillary shape/configuration; *PCD III* marked dilatation of calices and pelvis, pelvic axial width usually >10 mm with flattened papilla and rounded fornices but without parenchymal thinning; *PCD IV* gross dilatation of entire collecting system and thinning of renal parenchyma; *PCD V* used in some places to communicate an extreme PCD IV with only thin, membrane-like residual renal parenchymal rim

Additionally, referring to the previously published recommendations, we have taken subsequent steps to identify and then address some of the missing entities and procedures:

1. Imaging in anorectal and cloacal malformations in early childhood, which is a complex task and should be performed in a standardised fashion in dedicated specialised centres.
2. The procedural recommendations for a distal (fluoroscopic or sonographic) colostogram and for pelvic MRI in anorectal, cloacal and other genital malformations, respectively. These procedures are less commonly performed but still should be carried out in a standardised fashion.
3. The group also recommends an imaging algorithm in childhood ovarian torsion.
4. Finally, as a consequence of the latest effort to create a standardised terminology glossary for most paediatric uroradiologic terms, in order to be consistent with our terminology and considering the new American consensus classification proposal for congenital urinary tract dilatation conditions, our existing grading system for *hydronephrosis* in neonates and infants had to be renamed and now is called *pelvicalyceal distention/dilatation (PCD)*, still adhering to the same criteria and grades 0-V as a description for the collecting system appearance on a single (US) investigation (Fig. 1).

As always, the recommendations are based on a thorough literature search and discussion within the group. In addition, experts in the field were consulted -- not only from the field of paediatric radiology but also including referring clinicians such as paediatric urologists, paediatric surgeons and paediatricians. As with many conditions in childhood, there is a paucity of evidence available in the literature -- thus the recommendations are only in part evidence based whereas in other aspects they are based on expert opinion consensus statements. The proposals were discussed within the group via e-mail and internal group meetings. Furthermore, they were presented at the ESPR and the European Society of Urogenital Radiology (ESUR) annual meetings and discussed in open sessions. All comments and suggestions were considered before the final version of the proposed recommendation was developed.

### Imaging in anorectal and cloacal malformations

Anorectal malformation is a group of congenital anomalies involving the distal anus and rectum. The prevalence is around 1 in 5,000 newborns, with a male predominance. There is a high association (up to 70%) with other congenital malformations that often affect the final prognosis and outcome. The cloacal malformation is the most complex form of anorectal malformations (around 10%) in which the urinary tract, the vagina and the rectum converge into a single common outflow tract (persisting cloaca) that opens in the perineum. Persisting cloaca is almost exclusively seen in phenotypic females and is quite rare with an incidence of about 1 in 50,000 newborns. Cloacal malformation should not be confused with cloacal extrophy (although it is sometimes associated), which is a severe congenital defect of the abdominal wall with bladder and hindgut exposure and possible division of the genitalia. Cloacal malformations represent a wide spectrum of defects. Vaginal obstruction and Müllerian duct anomalies are often present, as well as urinary tract anomalies and many other associated malformations (e.g., spinal cord/spine, cardiac or oesophageal malformations). There is also an association with various syndromes and genetic disorders (e.g., VACTER(L), caudal regression syndrome, Currarino triad, Trisomy 21).

There is an old classification for anorectal malformations (the Wingspread classification of 1984) that defines a low, a high and an intermediate type depending on the position of the rectal pouch; additionally, cloacal and other rare malformations are listed. This classification was considered insufficient and a new international classification was introduced (the international Krickenbeck classification of 2005). The new classification also addresses and lists the various types of fistulas found in anorectal and cloacal malformations (perineal/cutaneous, bulbar or prostatic rectourethral, rectovesical, and/or vestibular fistula, the cloaca, and – without fistula – the simpler anal stenosis). Moreover, cloacal malformation and its variants can additionally be categorized into incomplete and posterior cloacal malformation, and the complete cloacal malformation with urinary-cloacal or urinary-rectal communications (urethra-cloacal or vesico-cloacal communication, vaginal communication of the rectum or rectal communication with the cloacal common channel).

For the surgeon, cloacal malformations are also classified according to the length of the common channel in long and short types, with a cutoff of 3 cm. All these classifications have an impact on therapy: incomplete malformations may not require urgent intervention, high (and intermediate) anorectal malformations will need temporary diversion via a protective and decompressive colostomy (sometimes also urinary deviation and drainage of a potential hydrocolpos) and late definite repair (usually 1 month-3 months later), whereas a low anal atresia can be treated by a primary pull-through repair or – in a stenosis – just dilatation.

The diagnosis as such is usually straightforward by clinical inspection and digital palpation. The role of imaging is:

1. First, to give an anatomical overview of the malformation and to assist treatment decisions by providing relevant data on the necessity of immediate surgical intervention. In all malformations with an occluded outlet tract, the length of the occluded segment and the position of the rectal pouch are crucial for immediate surgical decisions and the surgical approach – this (i.e. distance from perineum to pouch) can usually be answered by plain film and/or perineal US (see below).
2. Thereafter, to accurately characterise the complex anatomy of anorectal, cloacal and genitourinary, pelvic and perineal structures in order to allow for detailed surgical planning. Part of this is usually postponed and can be assessed after primary decompression/deviation later on.
3. Furthermore, early detection of associated anomalies is essential to prevent potential life-threatening complications.

**Fig. 2:**
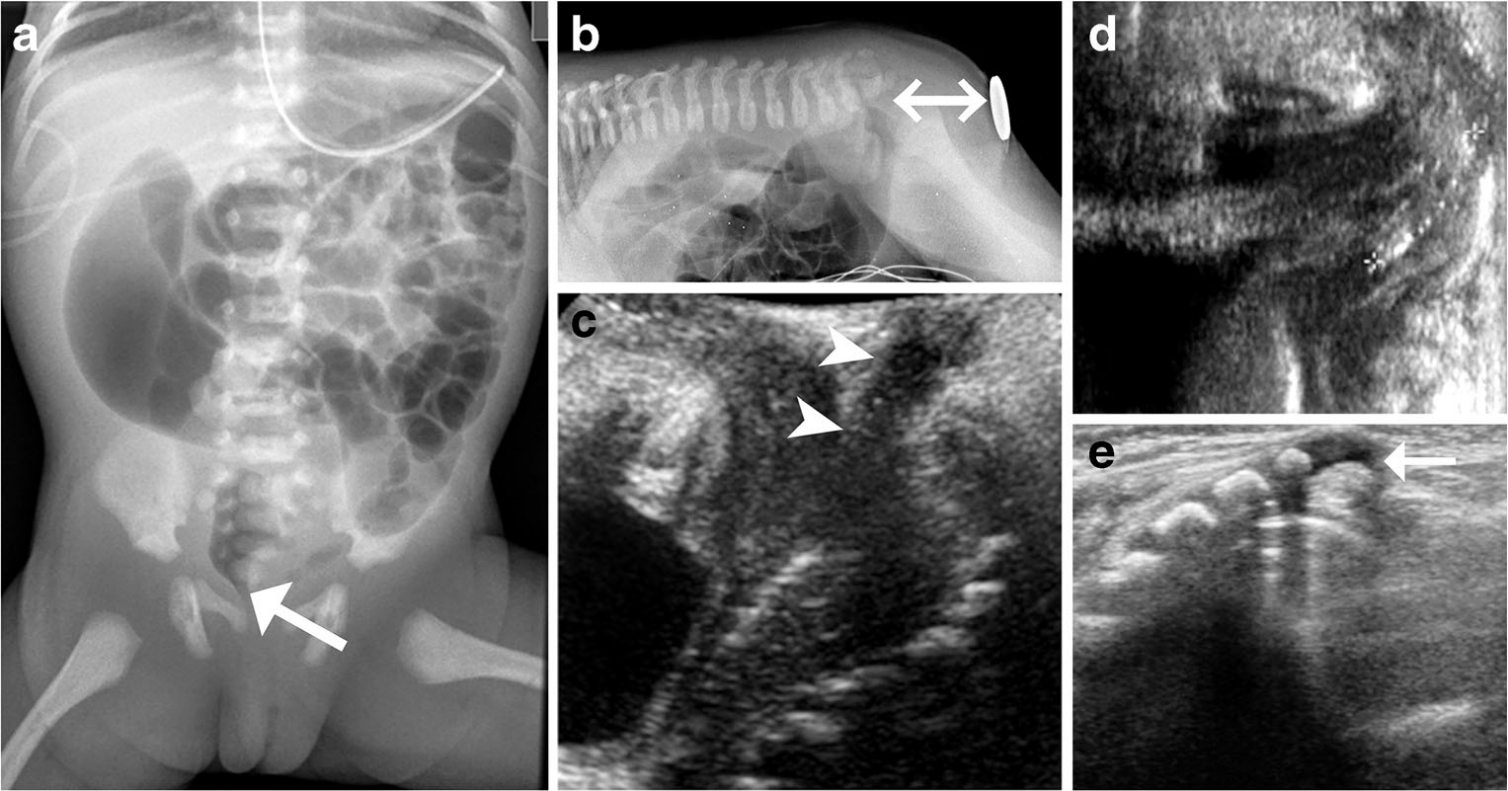
Initial imaging in anorectal and cloacal malformation. In a newborn girl with clinically evident anal atresia, an anterior-posterior abdominal radiograph (**a**) performed for initial overview and assessment of potentially associated (skeletal) malformations shows minor splaying of the sacral bodies and a high position of the rectal air (*arrow*). Lateral horizontal-beam prone radiograph (**b**) of the abdomen in a 2-day-old neonate with anal atresia. The area of the external anal opening is indicated by a marker and the distance from the air-filled rectal pouch to the anus (*double arrow*) can be measured if a ruler is added prior to taking the exposure. A newborn boy with anal stenosis: Transperineal US sagittal section (**c**) demonstrates the normal track of the narrow anal canal (*arrowheads*). Perineal US, sagittal section (**d**) in a neonate with anal atresia shows an air-filled fistula tract to the perineum (*between calipers*). A supplementary spinal US in prone position (**e**) with dorsal sagittal intonation shows a hypoplastic cartilaginous coccyx (*arrow*) with an unusually straight course in a 2-day-old girl with cloacal malformation

Plain films (chest, spine, abdomen – potentially a bottom-up view) and US are the most important initial imaging techniques (Fig. 2). A complete and comprehensive US examination of the pelvic cavity, including a perineal approach, is complemented by examining the entire urinary tract as well as the spinal cord.

**Fig. 3:**
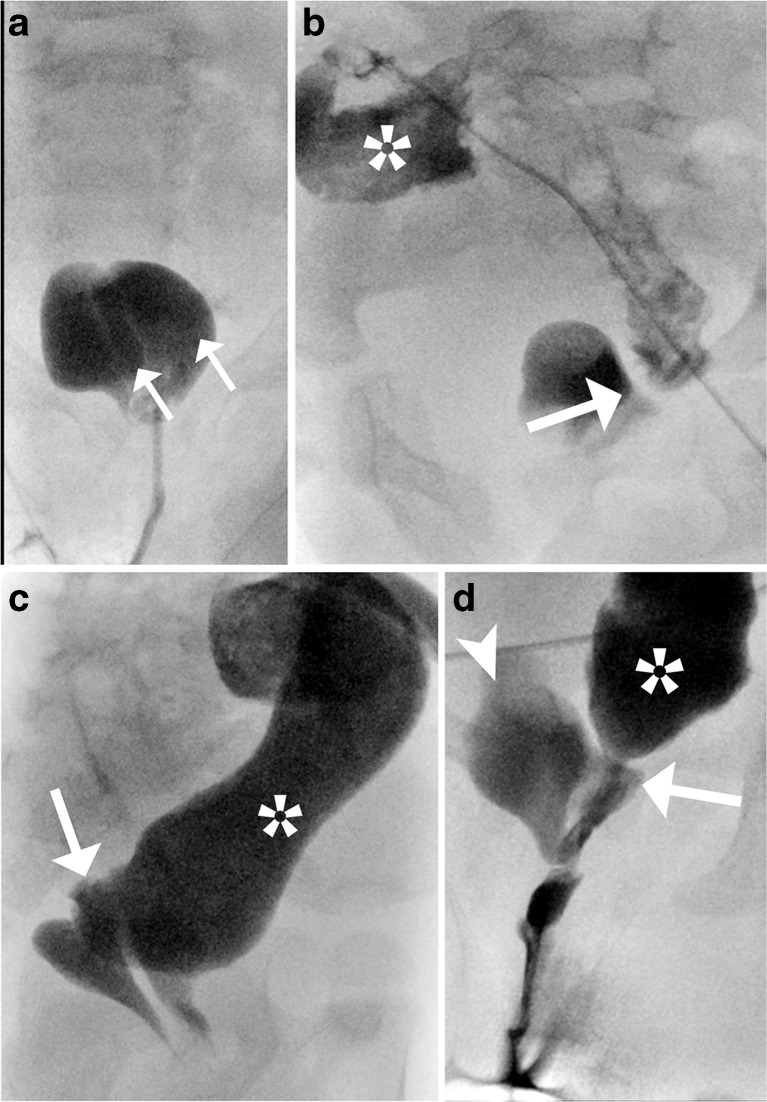
A newborn girl with anorectal malformation and fluoroscopic work-up. In the neonatal period, a distal retrograde fluoroscopic cloacogram (**a**) shows a duplex vagina (*arrows*) after catheterisation*.* A distal fluoroscopic colostogram (**b-d**) was performed later before definitive corrective surgery using the clostomy for opacification via catheter. The opacified colon (*asterisk*) is visualised draining into one of the two vaginas (*arrow*, **b**). After better filling of the colon (*asterisk*) via the colostomy, the detailed anatomy can be outlined, with a better visualisation of the fistula tract (*arrow*, **c**). At the end of the study (**d**), the urinary bladder (*arrowhead*), one vagina (*arrow*) and the rectal pouch (*asterisk*) with the connecting fistulas are opacified

Fluoroscopic genitography (or cloacogram), as the surgeon refers to it rather than to US genitography, can provide detailed anatomical characterization of the unpredictable course of the communications between the different pelvic structures (Fig. 3). A voiding cystourethrography should be attempted in the same session. If a colostomy is already present, a distal loop colostogram is performed beforehand, often avoiding the need for retrograde cloacal injection (Fig. 2). Some authors prefer to start by evaluating the genitourinary tract first, to hopefully force the contrast to go from the more sterile higher pressure urogenital compartment to the colon, and also to avoid the possibility that the opacified dilated colon obscures the genitourinary tract.

CT has also been advocated but should be avoided and reserved for selected cases, for instance preoperatively in complex cases if MRI is not available or possible.

**Fig. 4:**
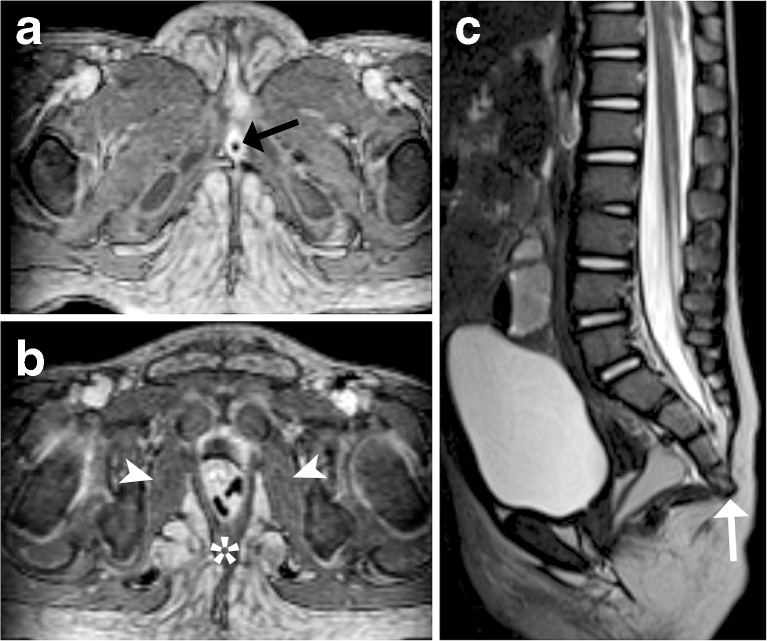
An MRI in a 1-year-old girl with cloacal malformation. (Axial T1-weighted turbo spin echo (**a,b**) for assessing the pelvic floor. A hypoplastic sacrum (*arrow*) is depicted in the sagittal steady-state free precession image (**c**)

**Fig. 5:**
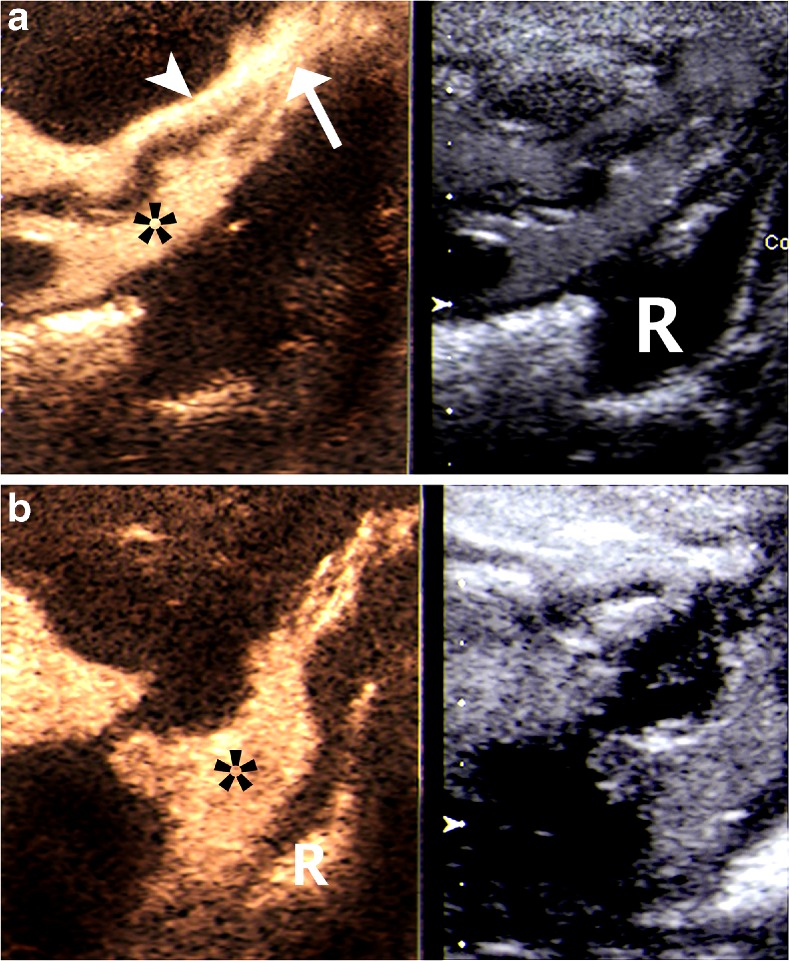
A newborn girl with cloacal malformation. Contrast-enhanced dynamic US genitography with perineal sagittal access is shown in a split dual image display with non-enhanced (left) and enhanced sonogram (right). **a** Contrast-filled urethra (*arrowhead*) and vagina (*asterisk*) with the respective common distal channel (*arrow*). The rectum is still filled with clear fluid (saline) from the earlier phases of the investigation (with saline instillation through the common external orifice). **b** At a later phase, there is US contrast agent both in the vagina (*asterisk*) and in the distal rectum (*R*)

MRI is the preferred method for cross-sectional imaging and MR machines with necessary equipment and protocols should be available in all referral centres for cloacal malformations. Detailed anatomical characterization by MRI can be difficult to achieve in small patients; the use of high-resolution T2-weighted 3-D MRI sequences and prior cloacal retrograde saline filling may then be helpful. Prior to definite surgical reconstruction, pelvic MRI can help define the position of the rectal pouch and the presence and developmental grade of the sphincteric muscle complex as well as give an overview of the entire often complex pelvic tracts (Fig. 4). Spinal MRI has a definite role in evaluating vertebral and spinal cord anomalies and MR urography can be advantageous for assessing associated urinary tract anomalies. It should be noted that all patients must be evaluated individually and imaging must be adapted to the clinical situation. Detailed anatomical characterization of the malformation should optimally be performed in the dedicated paediatric centre where the infant will be treated, with both paediatric radiologists and paediatric surgeons working in close relation. Additionally, contrast-enhanced (3-D) US may hold promise for the future (Fig. 5).

The proposed choice and timing of the imaging modality with respect to treatment options for anorectal and cloacal malformations in neonates and children have been extensively discussed.

**Fig. 6:**
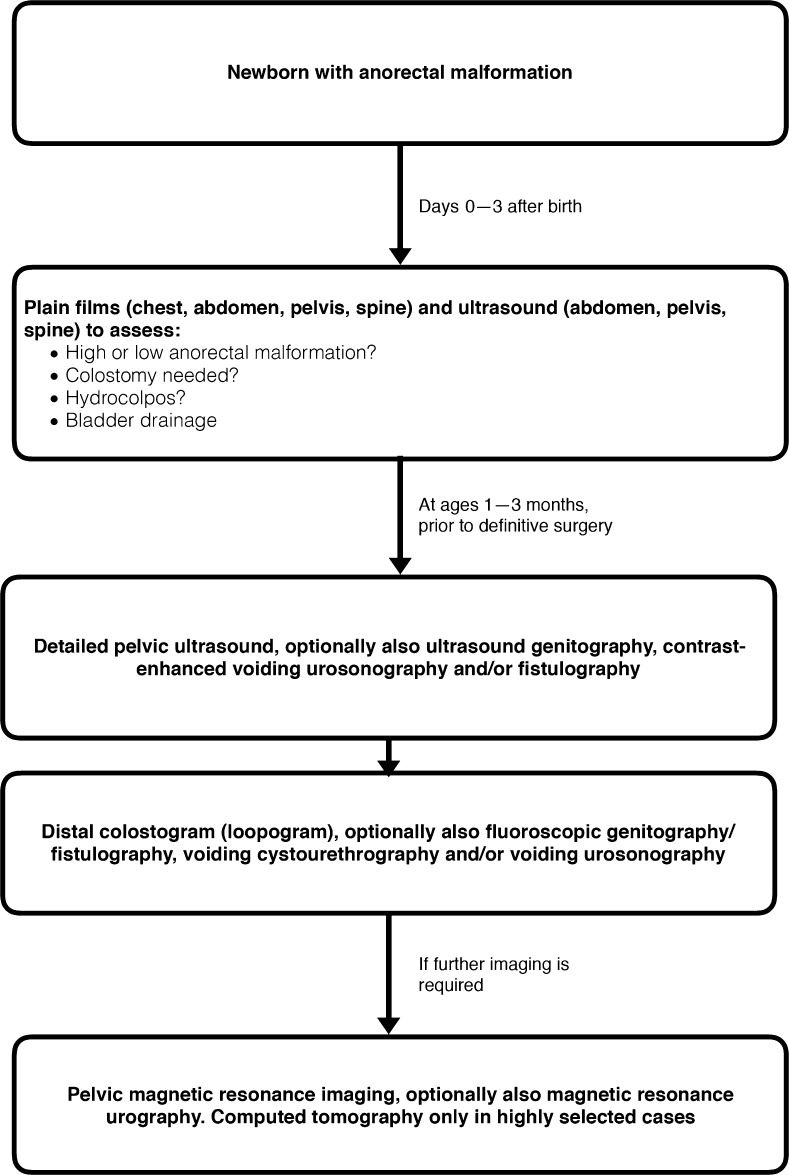
Imaging algorithm in newborns and infants with anorectal and cloacal malformations

The imaging algorithms for the delayed work-up differ from the initial diagnosis where US and plain film are often sufficient; this secondary work-up consists of fluoroscopy (colostogram, genitography/voiding cystourethrography) and MRI, which have become valuable and indispensable tools (Fig. 6).

### **Procedural recommendation – distal colostogram** (Fig. 7)

The purpose of performing a distal colostogram (loopogram) is to assess the distal colon itself (length and caliber), to determine the distance from the blind rectal pouch to the anal dimple, and to detect fistulas as well as to precise and characterize them (i.e. connecting structures, the course, the length, the caliber and the dynamic draining from the rectum into the vagina or the urethra). This filling of the distal colon via the colostomy for outlining the detailed anatomy is usually performed after enteric diversion in anorectal and cloacal malformations. Other specific conditions (for example, after bowel perforation or colonic atresia or Hirschsprung disease) are usually case-based depending on the underlying clinical scenario.

**Fig. 7:**
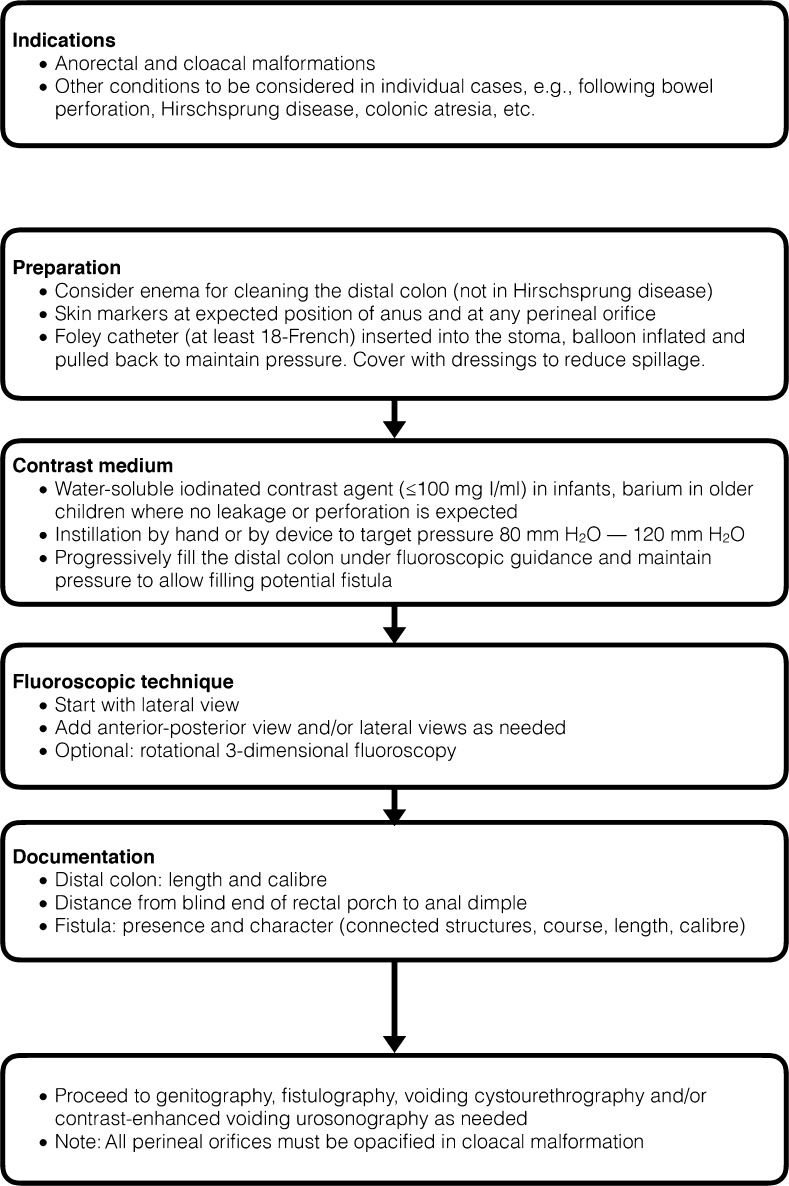
Procedural recommendation for distal colostogram (loopogram)

For preparation, one may consider previous distal colon cleaning with a conventional enema via the stoma or the anus (as opposed to Hirschsprung disease where colonic cleaning is not performed in order to avoid misdiagnosis by obscuring a transition zone). In anal atresia, skin markers are placed on the expected position of the anus and existing perineal external orifices are marked either by skin marks or by catheters. A Foley catheter (≥18 French) or a (fine) feeding tube is inserted into the stoma (sometimes a balloon is inflated and pulled back to maintain pressure). Otherwise, bandages can be considered to avoid spillage.

For fluoroscopy, water-soluble iodine contrast medium at a concentration of 100 mg iodine per milliliter or less is instilled in neonates and infants; barium can be used in older children if there is no suspicion of perforation or leakage into the peritoneum. The contrast agent is instilled by hand injection or by an infusion drip at 80- to 120-cm water pressure. A relatively high pressure should be maintained throughout the investigation to allow for depiction of potential fistula. Using pulsed fluoroscopy with last image hold technique, the distal colon is progressively filled and observed. One usually starts with the lateral view and then adds frontal views or additional oblique views as needed to document the findings. Sometimes a rotational 3-D fluoroscopy may further pinpoint the exact anatomical situation; if other structures cannot be properly outlined, these need to be identified by tubes and potentially by additional filling. Finally, a voiding cystourethrography and/or genitography must be added as needed if not performed previously, depending on the underlying condition. Particularly in cloacal malformation, all perineal orifices must be opacified and followed to the connecting structures.

For US, a similar approach can be used -- using saline as a contrast medium or a diluted US contrast agent solution (concentration as used for contrast-enhanced voiding urosonography, usually with 0.1–0.5% concentration). For proper sonographic access, a bladder catheter should be placed and the bladder filled, not only for anatomical identification but also to avoid obscuring deeper pelvic areas due to limited sonographic access. A perineal approach is mandatory for identifying distal tracts and fistula particularly during voiding. As for fluoroscopy, US contrast-enhanced genitography/contrast-enhanced voiding urosonography can be added (see earlier procedural recommendations) (Fig. 5).

### Procedural recommendation – pelvic MRI in cloacal and anorectal malformations (Fig. 8)

**Fig. 8:**
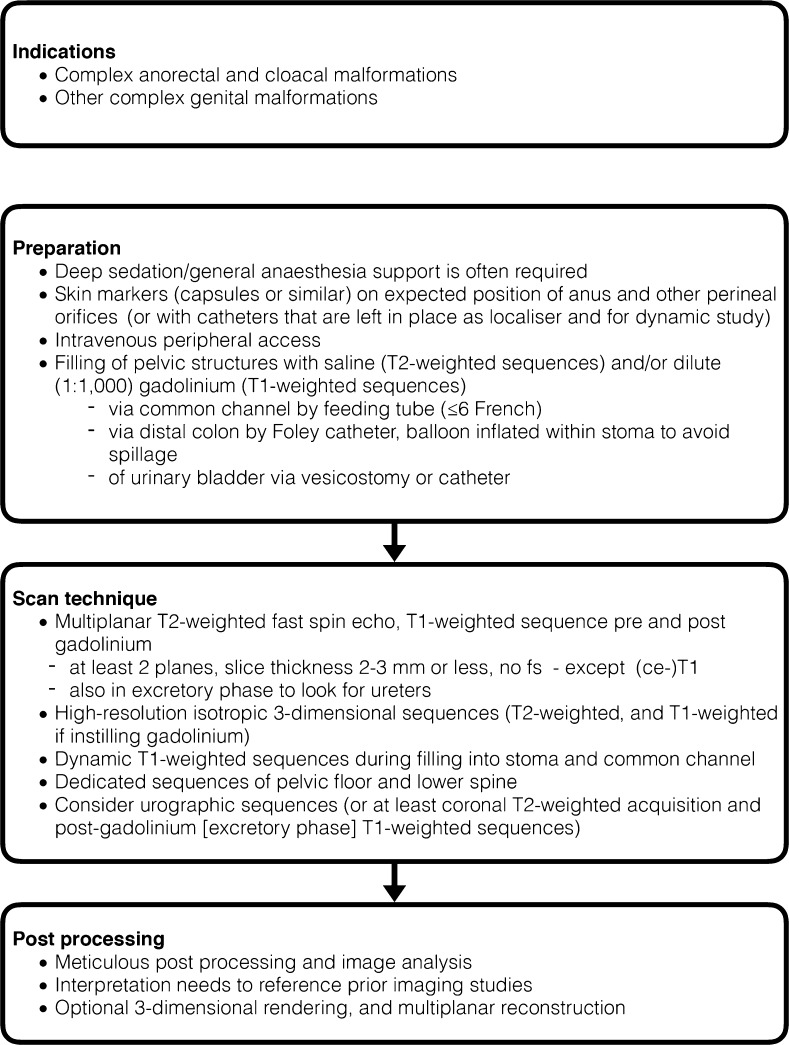
Procedural recommendation for pelvic MRI in anorectal and cloacal malformations

Due to restricted resolution and sedation needs with no urgent therapeutically relevant queries, pelvic MRI usually is performed not in the neonatal age but at a later stage when the pull-through procedure and closure of the colostomy are being scheduled. The procedure usually is performed in deep sedation or general anaesthetic support as the feed-and-wrap approach often is not sufficient to allow for the relatively long examination without motion while filling all the relevant pelvic structures; furthermore, it doesn’t always work in slightly older infants. When using adequate multi-channel coils and tailored sequences, 1.5-Tesla MRI systems are sufficient. While 3-Tesla scanners may offer a better depiction of pelvic muscles, more artifacts may be encountered. Skin markers (e.g., vitamin E capsules or similar) are placed on the expected position of the anus; other perineal orifices are marked as well. A peripheral intravenous line should be in place. For filling of the pelvic structures, a feeding tube (≤6 French) is placed in the common cloacal channel. The tube needs to be fixed to remain stable throughout the examination, both as a localizer as well as for a later dynamic study. The distal colon is filled by a Foley catheter potentially with an inflated balloon to avoid spillage. The urinary bladder is filled via a bladder catheter or a vesicostomy, depending on the given circumstances. Filling of the various structures can be performed with saline and/or diluted contrast agent; the bowel usually is filled with saline and nicely visualized in T2-weighted images. To better delineate a cloacal or vaginal structure again, saline or a dilute gadolinium suspension (1:100 to 1:1,000) can be instilled, which then can be imaged using T2- and/or T1-weighted sequences, respectively; concentration may be varied for filling of different structures. The urinary tract can usually be opacified in the late phase of a MR urography using an intravenous gadolinium application route, thus granting a physiological filling of the urinary tract including also the distal ureters from the kidneys and also allowing for assessment of the respective dynamic changes. Otherwise, the urinary bladder can be filled with dilute gadolinium as detailed above.

The technique consists of multiplanar imaging (at least in two planes) at a slice thickness of usually ≤2 mm acquiring T2-weighted spin-echo sequences and fat-suppressed T1-weighted images before and after gadolinium. Additionally, a high-resolution isotropic 3-D MR sequence in T2 weighting and (after contrast) with T1 weighting may be helpful and will allow for multiplanar reconstructions and 3-D navigation. Dynamic high-resolution (3-D) sequences similar to those used for MR urography or MR angiography could be used during the active filling into the stoma and the common channel as well as in the late phase of the MR urography for proper depiction of dynamics and the ureters; the pelvic floor and its muscles should always be included as well as the distal spine and spinal cord (Figs. 4 and 8). Respective meticulous post-processing is obviously mandatory and the interpretation usually is done comparing all other imaging results, e.g., from US or fluoroscopy.

### Imaging in childhood ovarian torsion

Ovarian and/or adnexal torsion is defined as complete or partial torsion of the ovary on its ligamentous supply, which eventually compromises the ovarian circulation. It is referred to as adnexal torsion if the fallopian tube is twisted along with the ovary; the rare (isolated) tubar torsion can also be encountered.

Ovarian/adnexal torsion is relatively rare in childhood (an estimated incidence of 4.9 per 100,000 females ages 1 year—20 years), but it is an important differential diagnosis in girls with abdominal pain. Ovarian torsion can also be encountered in neonates (potentially also prenatally), usually associated with ovarian cysts. Compared to testicular torsion, ovarian and adnexal torsion has a significant longer delay before clinical presentation and eventual diagnosis and, consequently, a more delayed treatment. Symptoms as well as laboratory tests are often nonspecific and it is often difficult to discern the clinical presentation from other causes of lower abdominal pain.

Ovarian torsion occurs much more commonly after menarche, but it may occur at all ages. Known risk factors are ovarian masses and cysts, reproductive age, pregnancy and previous ovarian torsion. The reason and mechanism for ovarian torsion is somewhat unknown; typically, the ovary and the fallopian tube twist around both the suspensory and the ovarian ligaments. A mobile and enlarged ovary predisposes to torsion whereas the elongated ovarian ligament in pre-menarchal girls and neonates that would permit excessive movement is only hypothesized. Ovarian torsion is more common on the right side, maybe because there is more space on the right side with the mobile caecum and ileum in contrary to the fixed and often filled sigmoid. Alternatively, the reported higher incident of right-side torsion might be secondary to a better detection rate, when looking for suspected appendicitis. Ovarian torsion eventually leads to occlusion of the vascular supply and causes a hemorrhagic infarction with necrosis. Experimental studies have shown that viable ovaries can be found up to 24 h with a complete necrosis after 36 h, whereas clinical reports show salvaged ovaries up to 72 h after the onset of symptoms. This may be due to the dual blood supply, which is probably beneficial, and may in part additionally be explained by partial torsion and intermittent detorsion. The effect of reperfusion injury on both the ipsi- and the contralateral ovary is unknown; however, gradual detorsion is shown to be beneficial in experiments. The long-term effect on fertility is unknown.

Clinically acute severe pain on either side of the abdomen or the pelvis, often with an intermittent character, is common. Nausea and vomiting, fever and/or a palpable abdominal mass may be present. Other symptoms include peritoneal pain and gastrointestinal or urinary tract symptoms. But there is no single symptom or sign that can confirm or preclude ovarian torsion. Often nonspecific biomarker inflammatory responses can be observed such as leukocytosis, elevated C-reactive protein, and increased IL-6 and CD64. In patients post menarche, ectopic pregnancy is considered (and potentially ruled out by looking at the level of human chorion gonadotropin) as well as ovarian tumors looking at additional tumor markers, e.g., alpha-fetoprotein.

**Fig. 9:**
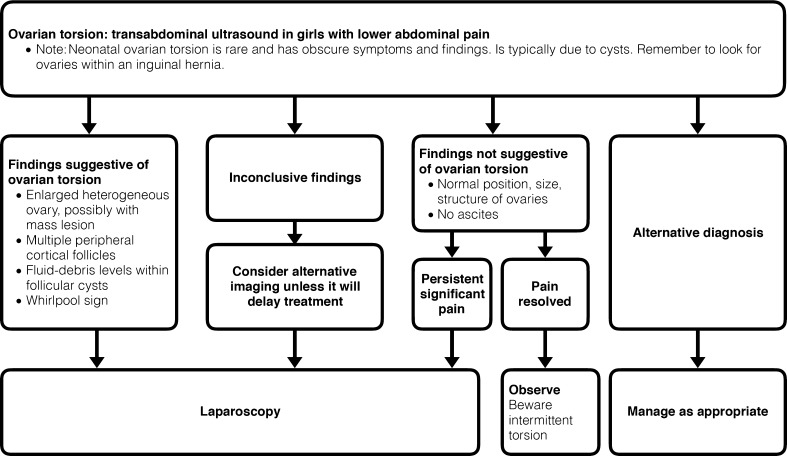
Recommendation for an imaging algorithm in childhood ovarian torsion

**Fig. 10:**
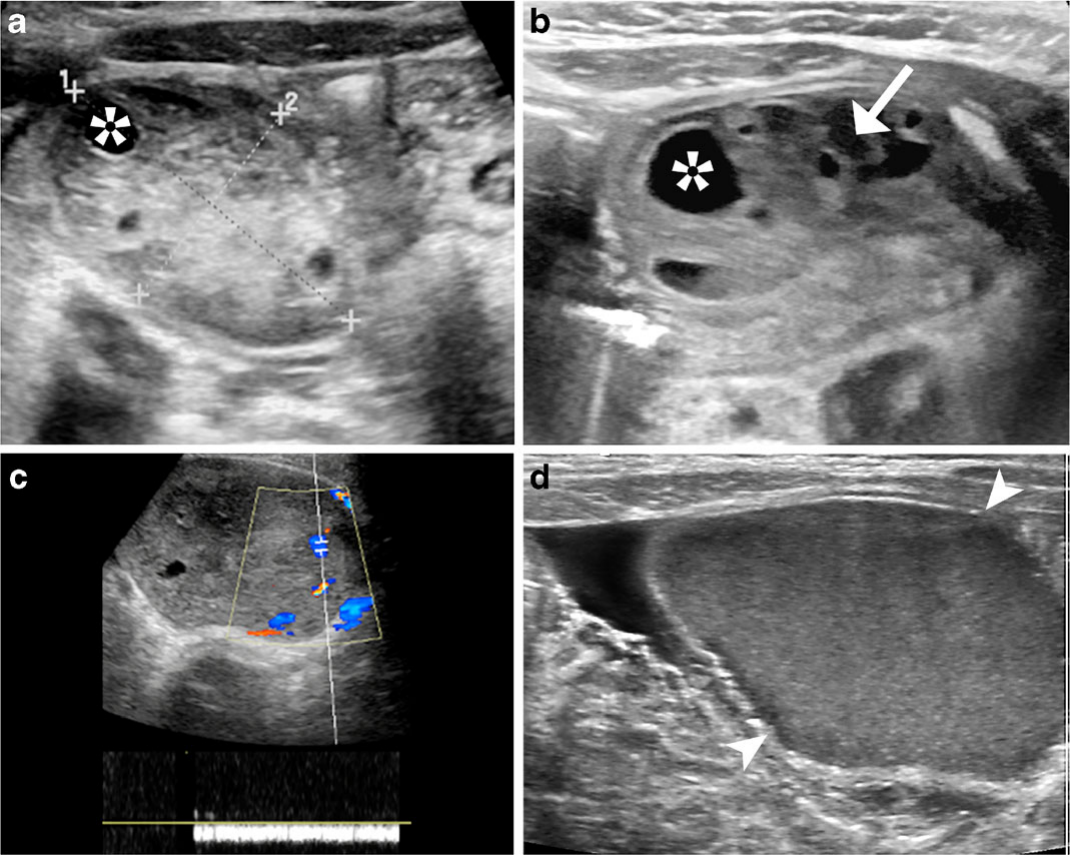
Typical US findings in childhood ovarian torsion. **a** The initial US of the lower abdomen in a pre-pubertal girl shows an enlarged ovary (*calipers*) with peripheral follicles (*asterisk*), consistent with an ovarian torsion. **b** Follow-up some days later in the same girl as in (**a**) shows cystic ovarian compartments (asterix) with sedimented echoes (arrow) indicating progressive hemorrhagic infarction. **c** US of the lower abdomen with a linear transducer performed for lower quadrant pain in a pre-pubertal girl depicts an enlarged, partially cystic ovary with stromal vessels on color Doppler (venous flow pattern) indicating that this probably is an ovarian tumor (with or without partial torsion). **d** US of the lower abdomen in a newborn girl. Axial section with a linear transducer shows an ovarian cyst (*arrowheads*) with internal echoes probably after hemorrhage. Even if large, this complicated neonatal ovarian cyst vanished without symptoms spontaneously within some months

The first imaging procedure to assess suspected childhood ovarian torsion is usually an immediate US scan, which allows for assessment of the ovaries as well as other potential causes of lower abdominal pain (Fig. 9). In children, a sufficiently filled bladder is very helpful – one should consider filling the bladder if ovaries cannot initially be depicted. On US, the most striking finding is an enlarged ovary with increased echogenicity typically with small peripheral cysts that exhibit echoes and sometimes layered sedimentation due to hemorrhagic infarction (Fig. 10). Displacement of the ovary and the uterus to the ipsilateral side is common. US should be performed using a transducer with the highest feasible frequency (in neonates 18–10 MHz, in infants 5–12 MHz, in older girls usually frequencies range between 2 and 8 MHz). Harmonic imaging can be helpful and a linear transducer will improve depiction of the typical small periphery follicles. Both ovaries and the uterus as well as the adnexal structures and the vascular supply should always be assessed, although there is a poor correlation of color Doppler findings with the diagnosis, particularly for ruling out ovarian torsion due to the ovary’s dual blood supply. A typical and diagnostically decisive sign is the twisted pedicle with a whirlpool/spiral sign of the ovarian pedicle. Free fluid in the pelvis is often seen. An ovarian or para-ovarian mass may be present, and is sometimes the cause of the torsion (i.e. a large cyst or an ovarian tumor; Fig. 10). The only proof for torsion by (Doppler) sonography is the presence of a whirlpool sign combined with lack of visible/depictable circulation, whereas the absence of depictable Doppler signals alone cannot predict ovarian torsion and the presence of Doppler signals in an otherwise abnormal ovary does not preclude torsion. Nevertheless, Doppler sonography cannot predict viability.

**Fig. 11:**
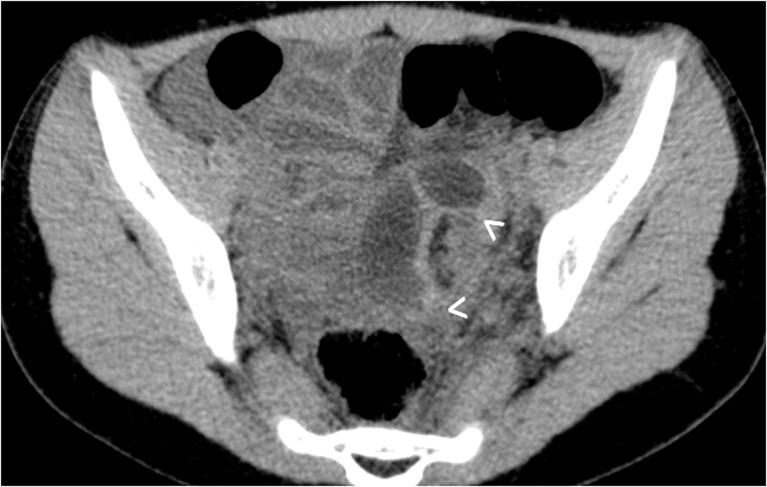
Non-enhanced CT, axial section, performed in a 12-year-old girl for unclear abdominal pain: A twisted left fallopian tube can be visualised (*arrowheads*)

**Fig. 12:**
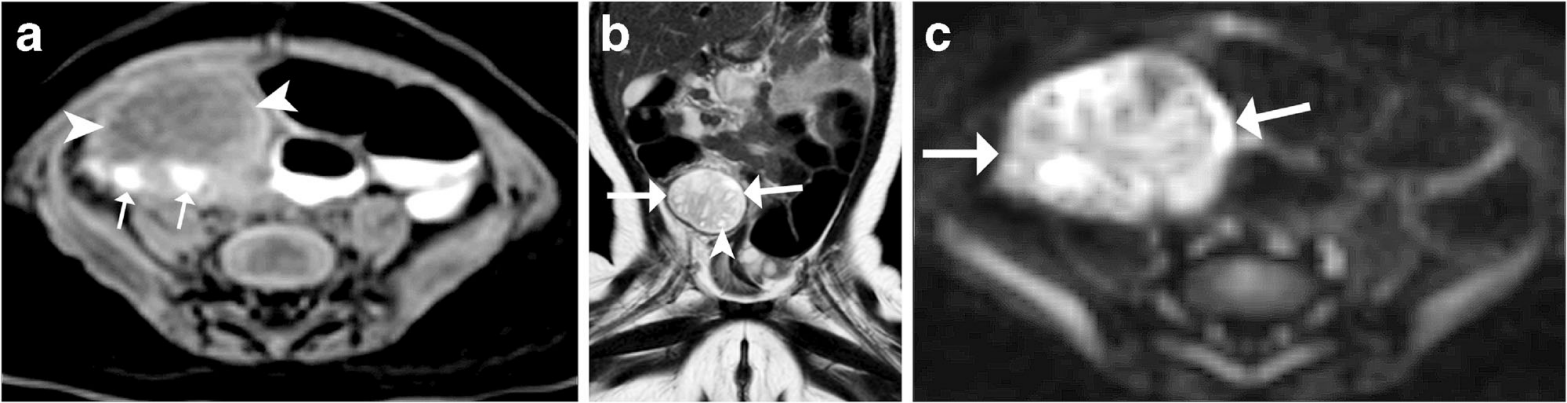
Typical appearance of ovarian torsion on MRI. in a 4 month old girl **a** Unenhanced axial T1-weighted fat-suppressed acquisition demonstrates hemorrhagic peripheral follicles (*arrows*) in the swollen and enlarged right ovary (*arrowheads*). **b** Coronal T2-weighted image shows the enlarged right-side ovary (*arrows*) with small peripheral follicles (*arrowhead*). **c** Axial diffusion-weighted image at b=1,000 demonstrates the diffusion impairment, inhomogenously distributed throughout the enlarged affected right ovary (*arrows*)

Other imaging methods, preferably MRI, may be used to assess ovarian torsion, but usually as second-line methods. However, these should only be used if they can readily be performed without any further delay. Nevertheless, the respective findings need to be known in case ovarian torsion is encountered on a MRI (or CT) performed for other queries. The findings are similar to the ones described for US: One will see ovarian enlargement -- often mass-like, there can be a reduced or rim-like contrast enhancement, surrounding fat infiltration and often free fluid or signs of hemorrhage (Fig. 11). The uterus is usually deviated towards the affected ovary. Multiple peripheral follicles are often depicted with clear signs of edema (e.g., on MRI stromal hyperintensity on T2-weighted images), a twisted vascular pedicle may be seen (whirlpool sign or peak formed margin of the ovary) and the fallopian tube is thickened and hyperintense on T2-weighted images. On diffusion-weighted imaging, restricted diffusion may be present (Fig. 12). First reports suggested that diffusion-weighted imaging can predict salvageability of the affected ovary; however, this is not yet confirmed.

The management is immediate, often conservative (i.e. the ovary is not removed) -- usually laparoscopic - surgery with detorsion and follow-up imaging, as underlying malignancy is rare. It needs to be noted that detorsion does not increase the risk of pulmonary embolism as previously suggested.

In neonates, the clinical presentation is similar, but often even more obscure or silent, and the most common predisposition for neonatal or peri−/prenatal ovarian torsion is an ovarian cyst (or other mass lesions such as dermoids or rare congenital tumors). The management of these congenital or neonatal cysts, however, is still controversial, particularly if they are larger than 3–5 cm (varies among institution). Smaller cysts are usually observed to see if they reduce spontaneously with instructions to parents to carefully look for signs of possible torsion; some also just follow larger cysts sonographically as long as they do not develop relevant displacement effects or become clinically symptomatic. Again, usually US is the main and only imaging modality for diagnosis and follow-up (Fig. 10). MRI may be indicated on suspicion of an underlying malignancy, although rare, particularly for staging or for differential diagnosis of equivocal cystic lesions. Fluoroscopy also may be indicated (e.g., assessment of a suspected communicating bowel duplication cyst or a cystic component in a genital malformation).

## Conclusion

Two new imaging algorithms for rare but typical childhood conditions (cloacal and anorectal malformation and childhood ovarian torsion) have been presented during this task force session and procedural recommendations for two important though rarer procedures (distal colostogram and pelvic MRI) have been proposed. Furthermore, comments and updates on the ongoing work have been addressed with finalisation of terminology standards for paediatric uroradiology as first presented last year; however, a dedicated separate paper will be published after final feedback from all other subspecialties and groups involved. Nevertheless – in order to be consistent with new terminology – the task force’s old *hydronephrosis* grading system had to be renamed and now is called *pelvicalyceal distention/dilatation (PCD)* grading using the same criteria as before to describe the appearance of the collecting system on a single US examination.

As with all proposals, the ESPR abdominal imaging task force heavily relies on feedback and input from those who use them. We are grateful for feedback and comments, and encourage everybody to engage in these projects. Hopefully, based on standardisation of imaging procedures and imaging algorithms, future meta-analysis and multicenter research will help put updates of these proposals on more solid evidence. Until these updates become available, the group’s proposals will hopefully help improve patient care by providing guidance on how to proceed with these queries and by offering suggestions for standardising the respective examinations, still allowing for individual adaptation and respecting different local settings, options and needs.

Nevertheless, it will remain a task for paediatric radiology to make sure that the most useful, still economically feasible methods are available to children. Therefore, to establish a dedicated US service (with appropriate equipment and proper training and supervision that also can reliably deal with relevant paediatric conditions in emergency settings 24 h a day 7 days a week throughout the year) is a prerequisite for granting proper health care to paediatric patients and for implementing the suggested imaging algorithms.
